# Over 2.5 million COI sequences in GenBank and growing

**DOI:** 10.1371/journal.pone.0200177

**Published:** 2018-09-07

**Authors:** Teresita M. Porter, Mehrdad Hajibabaei

**Affiliations:** 1 Centre for Biodiversity Genomics & Department of Integrative Biology, University of Guelph, Guelph, Ontario, CANADA; 2 Great Lakes Forestry Centre, Natural Resources Canada, Sault Ste. Marie, Ontario, CANADA; University of Innsbruck, AUSTRIA

## Abstract

The increasing popularity of cytochrome c oxidase subunit 1 (COI) DNA metabarcoding warrants a careful look at the underlying reference databases used to make high-throughput taxonomic assignments. The objectives of this study are to document trends and assess the future usability of COI records for metabarcode identification. The number of COI records deposited to the NCBI nucleotide database has increased by a geometric average of 51% per year, from 8,137 records deposited in 2003 to a cumulative total of ~ 2.5 million by the end of 2017. About half of these records are fully identified to the species rank, 92% are at least 500 bp in length, 74% have a country annotation, and 51% have latitude-longitude annotations. To ensure the future usability of COI records in GenBank we suggest: 1) Improving the geographic representation of COI records, 2) Improving the cross-referencing of COI records in the Barcode of Life Data System and GenBank to facilitate consolidation and incorporation into existing bioinformatic pipelines, 3) Adherence to the minimum information about a marker gene sequence guidelines, and 4) Integrating metabarcodes from eDNA and mixed community studies with existing reference sequences. The growth of COI reference records over the past 15 years has been substantial and is likely to be a resource across many fields for years to come.

## Introduction

Cytochrome c oxidase subunit 1 (COI) marker gene or DNA barcode sequencing of animals from mixed communities and bulk samples has surged in usage [1]. COI metabarcoding is a scalable method that can take advantage of automated work-flows, improve throughput, and facilitate large-scale studies [2,3]. COI metabarcoding applications include diversity assessments for biomonitoring and conservation [4,5], detection of environmental gradients in ecology and forestry studies [6,7], and diet analysis [8,9].

COI metabarcoding leverages existing COI sequences in databases such as the Barcode of Life Data (BOLD) System as well as the International Sequence Database Collaboration (INSDC) between the National Center for Biotechnology Information (NCBI), the European Bioinformatics Institute (EMBL-EBI), and the DNA Data Bank of Japan (DDJB) [10,11]. Automated taxonomic assignment of anonymous COI metabarcodes from mixed samples depends on the availability of representative reference sequences for comparison. For taxonomic assignment of large batches of COI sequences from high throughput sequencing platforms, popular methods include the top BLAST hit approach or the naïve Bayesian COI Classifier [12,13]. Both of these methods rely on publically available COI reference sequences. Overviews of the taxonomic coverage of COI sequences in the NCBI nucleotide database have been published [13–15]. Previous work has focused on invertebrates and Insecta for freshwater biomonitoring in general and for Canadian freshwater biomonitoring in particular.

Past studies have looked at the ability of COI metabarcodes to identify taxa from mixed samples by using different taxonomic assignment methods [13,14]. A common misunderstanding among researchers new to the field is that all target taxa are present in the reference database for comparison. Most experienced researchers know this is false. When target taxa are missing from the reference database, users run the risk of making false positive and false negative taxonomic assignments [16]. The mis-assignment of a metabarcode to the wrong species with high confidence, because the target species is missing from the database has been called a false-positive assignment or over prediction in the literature [17]. False negatives also occur when there are gaps in the database leading to low confidence taxonomic assignments. The current study takes a step back to outline the type and quality of COI data contained in the NCBI nucleotide database and the implications for future work.

Here we focus on the current level of taxonomic and metadata annotation of COI sequences in the NCBI nucleotide database. We describe trends since the inception of COI barcoding and implications for COI metabarcoding going forward. We highlight two COI metabarcoding applications: 1) freshwater invertebrate biomonitoring, and 2) detection of endangered animal species as listed by International Union for Conservation of Nature (IUCN). We chose these examples to illustrate COI coverage for two very different metabarcoding applications. For freshwater benthic taxa, we anticipated good COI database representation, at least for North American genera [5,15]. For more remote and tropical regions, especially at the species level, we expected database representation to be less complete [15,18]. We also hoped to illustrate the potential for COI metabarcoding of endangered animal species based on encouraging results from previous COI barcoding studies of Bovidae, antelopes, and placental mammals [19–21]. Metadata analysis shows that the COI records in the NCBI nucleotide database have increased substantially since the introduction of COI barcoding to the community and includes records with a global geographic distribution. In this high-level analysis, we highlight a few areas to improve COI sequence usability across studies: 1) Improving the representation of COI records from more diverse geographic regions, 2) Improving the cross-referencing of COI records in BOLD and the NCBI nucleotide database to facilitate consolidation and incorporation into existing bioinformatic pipelines, 3) Adherence to the minimum information about a marker gene sequence (MIMARKS) guidelines, and 4) Integrating metabarcodes from eDNA and mixed community studies with fully identified sequences from individual specimens.

## Bioinformatic methods

GenBank data was parsed using a combination of command-line and custom Perl scripts using BioPerl modules [22]. Tabular data was formatted using Python and plotted in R [23]. We use the terminology from Nilsson et al., (2005) and refer to taxa identified to the species rank as ‘fully identified’ and all other taxa as ‘insufficiently identified’ [24]. We also focused on NCBI nucleotide data deposited from 2003, the year COI barcoding was first introduced to the community, to present (2017) [25].

The names and taxonomic identifications for all Eukaryotes annotated to the species rank were retrieved from the NCBI taxonomy database using the Entrez query "Eukaryota[ORGN]+AND+species[RANK]" with an ebot script [Accessed November 3, 2017] [26]. Taxa were filtered according to the contents of the species field so that only fully identified taxa with a complete Latin binomial (genus and species) were retained. Entries that contained the abbreviations sp., nr., aff., or cf. were discarded. The remaining species names were formatted for use in the next query [*species list*]. For each year from 2003–2017 [*year*], records in the NCBI nucleotide database containing COI sequences were retrieved using the Entrez query "("CO1"[GENE] OR "COI"[GENE] OR "COX1"[GENE] OR "COXI"[GENE]) AND "Eukaryota"[ORGN] AND [*year*][PDAT]) AND [*species list*]” [2003–2016, accessed November 2017; 2017, accessed April 2018]. GenBank records were parsed, retaining information on year of record deposition and number of fully identified records. For fully identified records, sequence length as well as country and/or latitude-longitude fields were parsed.

We also assessed the number of high quality COI sequences that meet the standards developed between the INSDC and the Consortium for the Barcode of Life by looking for the BARCODE keyword in the GenBank record [11]. For each year from 2003–2017 [*year*], records in the NCBI nucleotide database containing COI BARCODE sequences were retrieved using the Entrez query "("CO1"[GENE] OR "COI"[GENE] OR "COX1"[GENE] OR "COXI"[GENE]) AND "Eukaryota"[ORGN] AND [*year*][PDAT] AND “BARCODE”[KYWD]) AND [*species list*]”. Fully identified and geotagged records were parsed as described above.

For our application example on freshwater biomonitoring, we retrieved a high-level list of relevant groups from Elbrecht and Leese (2017) to facilitate comparisons across studies [27]. Target freshwater taxa included: Annelida classes Clitellata and Polychaeta; Insecta (Arthropoda) orders Coleoptera, Diptera, Ephemeroptera, Megaloptera, Odonata, Plecoptera, and Trichoptera; Malacostraca (Arthropoda) orders Amphipoda and Isopoda; Mollusca classes Bivalvia and Gastropoda; and Platyhelminthes class Turbellaria. Within these groups there are likely to be non-freshwater taxa included, however, this method allowed us to quickly gauge the representation of freshwater taxa contained therein. These are also the same groupings often used to summarize results from COI freshwater biomonitoring assessments. A detailed look at specific freshwater taxa at finer taxonomic levels is beyond the scope of this paper and will be published elsewhere. For each freshwater target group we queried the NCBI taxonomy database for records identified to the species rank as described above. These taxon ids were concatenated and used to query the NCBI nucleotide database as described above. We assessed the representation of freshwater indicator taxa in the NCBI nucleotide database and level of annotation as described above.

For our application example on IUCN endangered animal species, we retrieved a list of endangered species names from http://www.iucnredlist.org from all available years (1996, 2000, 2002–2004, 2006–2017) filtering the results for native Animalia species [Accessed Dec. 12, 2017]. We excluded insufficiently identified species containing the terms ‘affinis’, ‘sp.’, or ‘sp. nov.’, leaving us with a list of 4,289 endangered animal species as well as 2,089 synonyms. We submitted this combined list of species names to the ‘NCBI Taxonomy name/id Status Report Page’ (https://www.ncbi.nlm.nih.gov/Taxonomy/TaxIdentifier/tax_identifier.cgi) and retrieved a list of 2,613 taxon ids. For each taxon id, we queried the NCBI taxonomy and nucleotide databases as described above.

To assess the number of COI records unique to the BOLD database compared with the NCBI nucleotide database, we also retrieved records from the BOLD Application Programming Interface (API) as well as from the data releases. Since the BOLD database contains records from several DNA barcode markers such as ITS rDNA for fungi and COI mtDNA for animals, it was necessary to target just the COI records. COI sequences were retrieved from the BOLD API (http://www.boldsystems.org/index.php/API_Public/sequence?) using the terms ‘marker = COI-3P|COI-5P&taxon = ‘ for each Eukaryote phylum except for Arthropoda which was queried separately for each class, and Insecta which was queried separately for each order to enable the download of complete files [Accessed Apr. 26, 2018]. Lists of Eukaryote phyla, Arthropoda classes, and Insecta orders were retrieved from the BOLD taxonomy browser (http://www.boldsystems.org/index.php/TaxBrowser_Home). COI records were also retrieved from the BOLD data releases (http://www.boldsystems.org/index.php/datarelease). All available releases of animal COI records up to and including Release 6.50v1 were individually downloaded and parsed. Note that the records retrieved from the data releases may not be as current as those retrieved through the BOLD API.

## Results

The dataflow and scripts used in this study are available from GitHub at https://github.com/terrimporter/COI_NCBI_2018.

### COI record growth in GenBank

**Fig 1:**
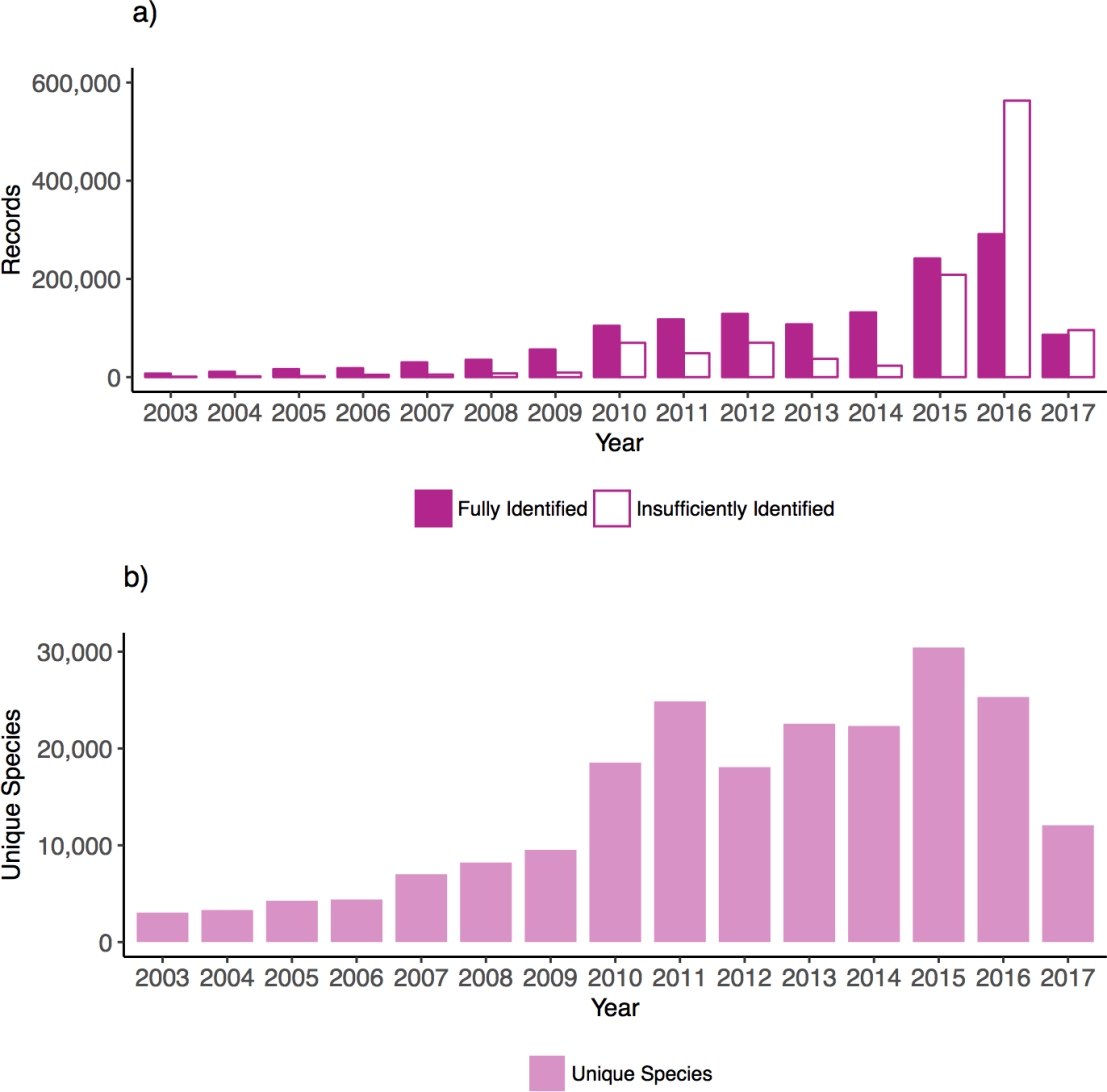
The number of Eukaryote COI records and unique species in the NCBI nucleotide database has grown since 2003. A) The number of records deposited from 2003 to 2017 (inclusive). B) The number of unique species that the fully identified records represent.

We show the growth of COI records in GenBank from the introduction of COI barcoding in 2003 to 2017 (inclusive). A total of 2,530,418 COI Eukaryote records were identified from the NCBI nucleotide database. Of these, 1,383,206 (55%) records were verified to be fully identified COI records. We express database growth as the geometric average of growth rates from year to year. The COI records deposited to the NCBI nucleotide database increased on average by nearly 51% per year (Fig 1). There were two especially large COI record depositions in 2015 (244,289) and 2016 (295,710). In 2017, 108,477 records were deposited and this is more in line with the number of records deposited in 2010–2014 (106,197–131,836). The number of insufficiently identified records has also grown by 66% per year compared with 46% per year for fully identified records. The number of unique species represented in records deposited in 2017 (12,069) is closer to the range from 2003–2009 (3,046–9,522) and is less than the range from 2010–2016 (18,071–30,429).

### Target COI taxa in the NCBI nucleotide database

**Fig 2:**
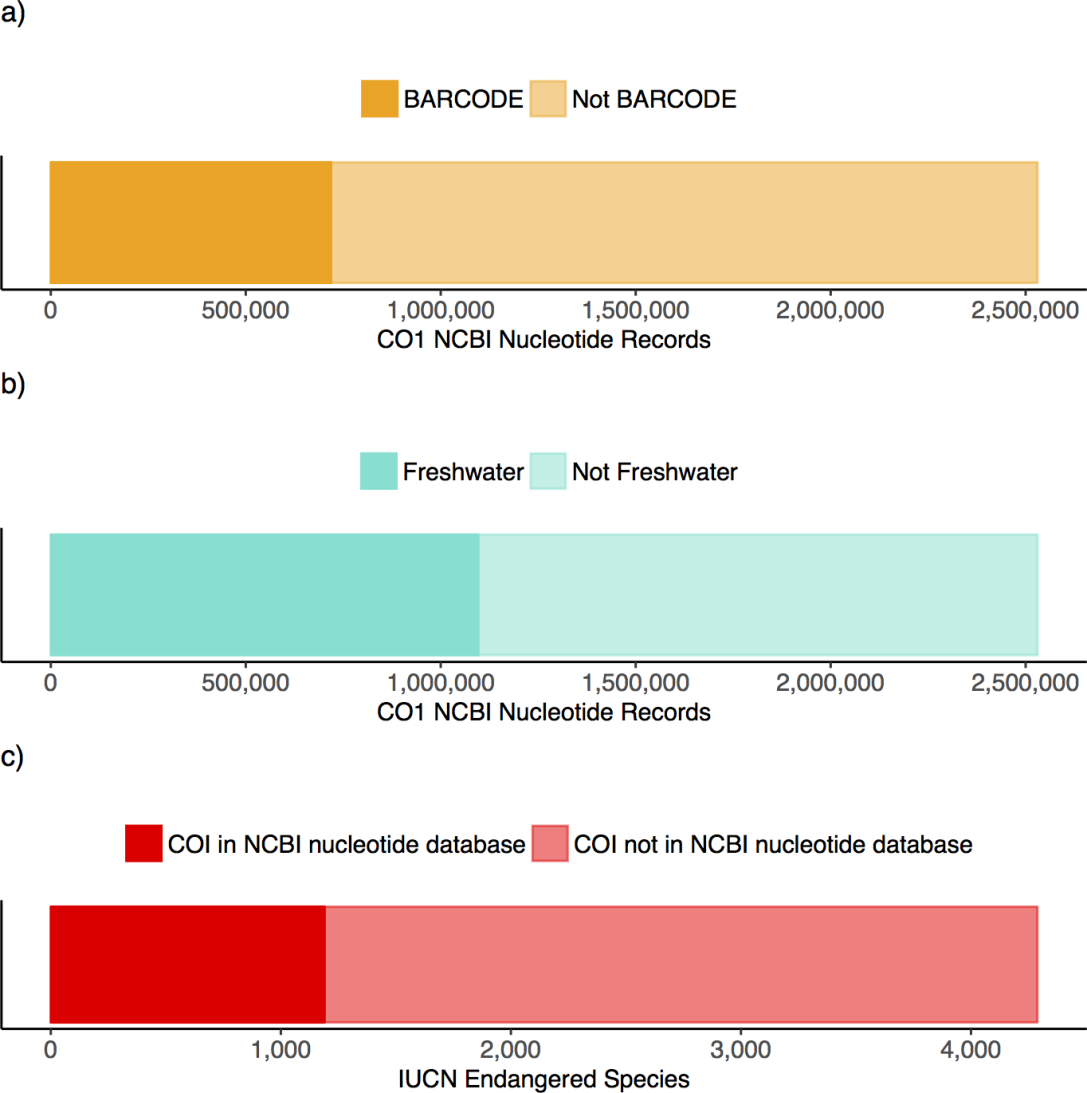
Summary of target taxonomic groups in the NCBI nucleotide database. A) The proportion of all Eukaryote COI records flagged with the BARCODE keyword. B) The proportion of all Eukaryote COI records that represent high-level freshwater biomonitoring target taxa. C) The proportion of IUCN endangered animal species that are represented by COI records.

When looking at the distribution of COI Eukaryote records in the NCBI nucleotide database we found that 718,814 (28%) are flagged with the BARCODE keyword indicating these meet the standards created in consultation with Consortium for the Barcode of Life [11] (Fig 2). 1,096,518 (43%) represent high-level freshwater biomonitoring taxa of interest. Records for freshwater taxa largely represent Diptera (true flies, 728,906), Coleoptera (beetles, 151,841), and Gastropoda (snails and slugs, 76,786) (S1 Fig). 1,190 (28%) of the IUCN endangered animal species have corresponding COI records in GenBank. A total of 11,934 NCBI nucleotide COI records correspond to IUCN endangered animal species.

We also show that the number of COI records deposited to the NCBI nucleotide database for specific groups of taxa (BARCODE, freshwater, endangered) has substantially grown from 2003 to 2017 (S2 Fig). The number of BARCODE records deposited increased on (geometric) average by 78% per year with 386 records deposited in 2004 and a total of 718,714 records by the end of 2017. The number of records belonging to high level freshwater taxa has increased by 52% per year with 3,217 records deposited in 2003 and a total of 1,096,518 records by the end of 2017. In both cases, the number of insufficiently identified records has also grown on average by 69% per year for BARCODE taxa and 71% for freshwater taxa. The number of records deposited that represent endangered species has grown on average by 27% per year with 15 records deposited in 2003 and a total of 3,217 records by the end of 2017.

### COI NCBI nucleotide record annotations

**Fig 3:**
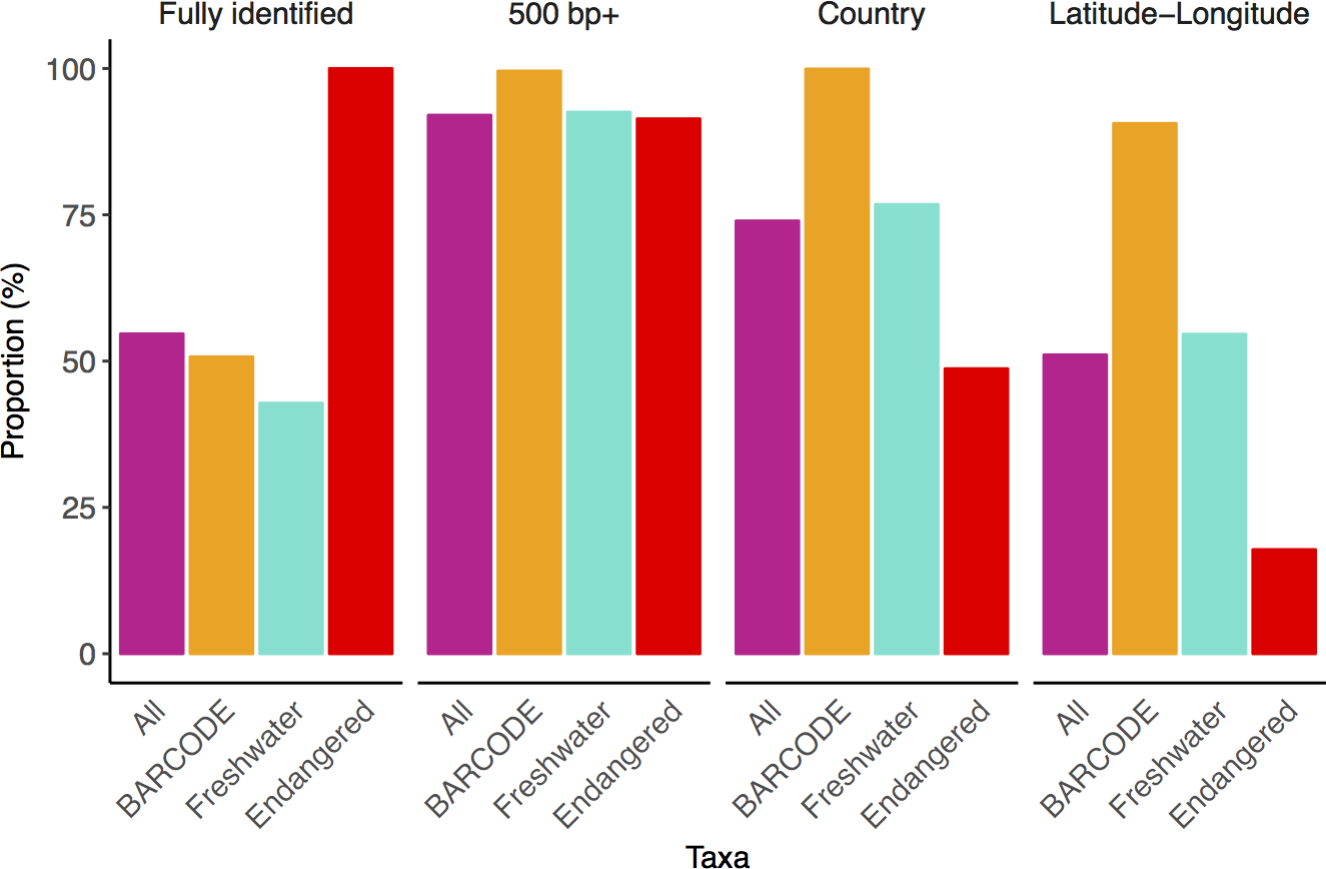
COI BARCODE records in the NCBI nucleotide database are well annotated. The first panel shows the proportion of records that are fully identified to the species rank. The remaining three panels show the proportion of fully identified records with good sequence length (500 bp +), country, and latitude-longitude annotations. ‘All’ refers to the complete set of COI Eukaryote records deposited to the NCBI nucleotide database from 2003 to 2017. BARCODE refers to the subset of records flagged with the BARCODE keyword. Freshwater refers to the subset of records that represent high-level freshwater biomonitoring taxa of interest. Endangered refers to the subset of fully identified records on the IUCN endangered animal species list.

Overall COI record annotation completeness was highest for BARCODE flagged records (Fig 3). The proportion of fully identified BARCODE records was 51% and similar to the level of fully identified records for All Eukaryotes and the subset of freshwater taxa. Nearly all of the fully identified BARCODE records had good sequence length (500 bp+) and were geotagged with country and latitude-longitude information as we would expect from such records. In contrast, the proportion of endangered species that were fully identified is 100% by default because we were searching for a specific list of endangered species. Records for endangered species were relatively incomplete with 49% that included country and 18% that included latitude-longitude data.

### Geographic distribution of COI records

**Fig 4:**
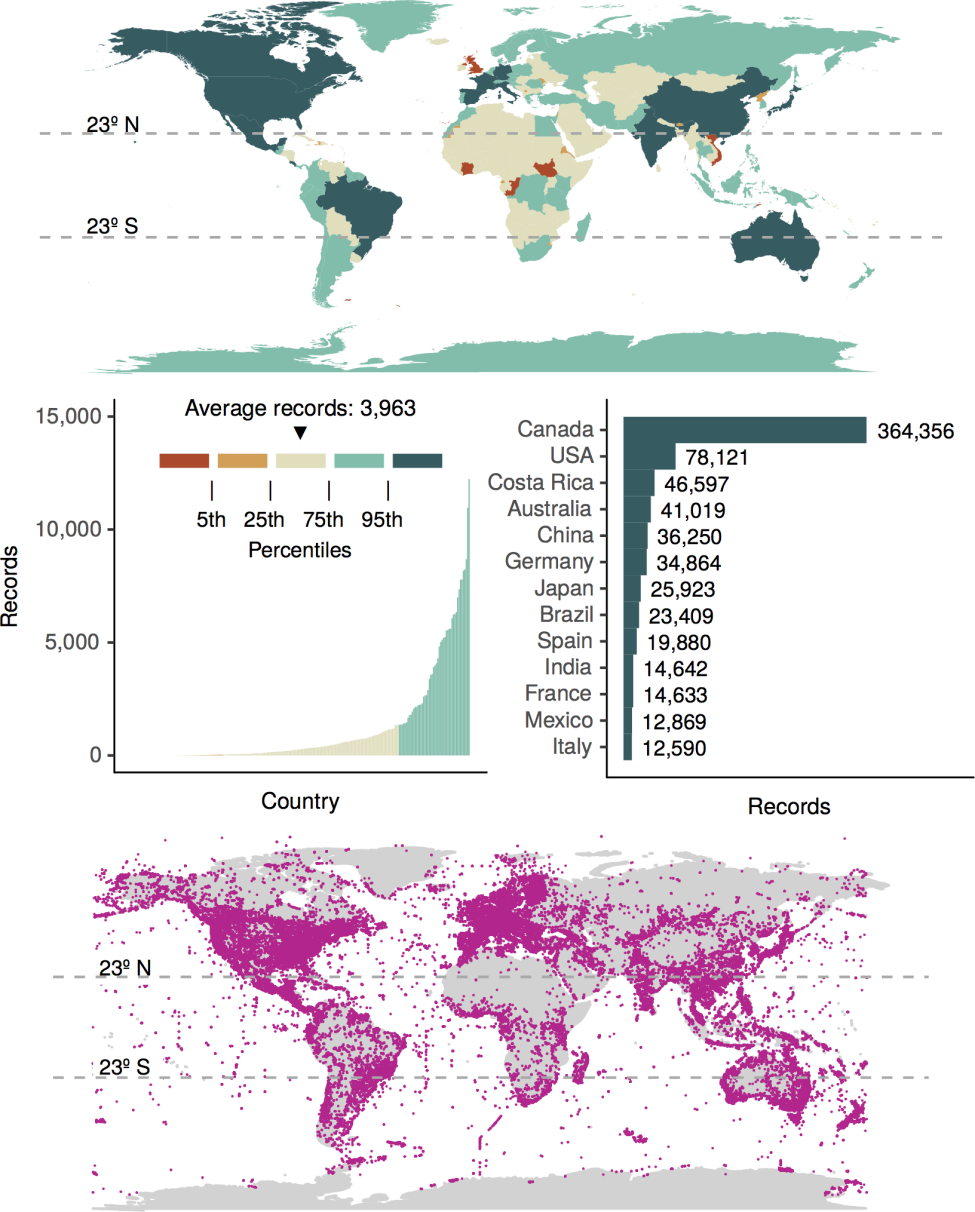
Worldwide distribution of fully identified COI Eukaryote NCBI nucleotide records. Top panel: The number of records per country, where this information was available, is shown. Middle panel: The number of records per country with the bottom 95% of countries shown on the left, and the top 5% of countries shown on the right. Bottom panel: Latitude-longitude data, where this information was available, are plotted as pink points.

Fully identified COI NCBI nucleotide records show a global distribution but are biased towards Canada (364,356) (Fig 4). There are nearly as many fully identified records with no country data provided (360,194). The 5 next best-represented countries are USA (78,121), Costa Rica (46,597), Australia (41,019), China (36,250), and Germany (34,864). Country annotation data are useful, but because of variations in spelling, as well as country borders and names that change over time, this can be a difficult metadata field to standardize across studies. Latitude-longitude data provide more resolution of COI record distribution within countries and are easier to combine across data sets but we found this data is often lacking in non-BARCODE COI records. Similar maps for BARCODE, freshwater, and endangered animal species are also provided (S3 Fig). BARCODE records disproportionately represent North American, European, and Australian sites, and globally the representation is better than for the freshwater or endangered species datasets. Freshwater records tend to represent European and North American sites with relatively less coverage of other continents. Endangered animal species records are relatively scant by comparison with the largest number of records coming from Europe, North America, and parts of Asia. In all datasets, records from Antarctica, Greenland, and most of Africa are sparse.

We retrieved a final set of 4,646,172 COI sequences from the BOLD API (Table 1). 15% (705,711) of these sequences were associated with a GenBank record flagged with the BARCODE keyword. 48% (2,238,104) had GenBank accessions that were not flagged with the BARCODE keyword. This set of the records seems to be either mined from GenBank and used to supplement the BOLD database, or they were BOLD records deposited to GenBank then subsequently suppressed because the record did satisfy the iBOL/GenBank early release agreement. The remaining 37% (1,715,362) of the records appear to be unique to BOLD.

**Table 1:** Distribution of COI records in BOLD.

BOLD Source	BOLD Record with GenBank accession + BARCODE keyword	BOLD Record with GenBank accession	Remaining BOLD Records	Total
BOLD API	705,711 (15%)	2,238,104 (48%)	1,715,362 (37%)	4,646,172
BOLD Data Releases	341,150 (13%)	896,150 (33%)	1,490,534 (55%)	2,727,834

A total of 2,727,834 unique records were retrieved from the BOLD data releases (Table 1). 13% (341,150) were associated with a GenBank record flagged with the BARCODE keyword. 33% (896,150) were associated with a GenBank record but did not contain a BARCODE keyword. Some of these records were found to contain iBOL details in the GenBank record COMMENT section, others were not indicated as being BOLD records in any way. 55% (1,490,534) of the records appear to be unique to BOLD. Whether or not a linked GenBank record was subsequently suppressed is not indicated here.

## Discussion

### Taxonomic representation

Based on existing work, we already knew that the best-represented taxonomic groups of COI sequences in the NCBI nucleotide database were Arthropods, followed by Chordata [13]. More specifically, the class Insecta, and the order Diptera contained therein, have the largest numbers of COI records in GenBank [14]. To assess whether the available reference data is sufficiently complete for real-world application of COI metabarcoding for freshwater biomonitoring, recent studies have addressed this point. A study by Curry et al. (2018) looked specifically at North American taxa expected to be recovered during freshwater biomonitoring using traditional methods and checked for their presence in GenBank and BOLD [15]. They found that ~ 61% of freshwater genera were associated with COI sequences in both databases. Either database alone, however, only provided coverage of ~51% of genera due to genera being present in only one database or the other. Practically, however, they found that ~95% of genera found in more than 1% of field samples were associated with COI sequences. In the current study, we found that ~ 43% of the COI records in the NCBI nucleotide database represent high-level freshwater taxa of interest for biomonitoring. Similar to Curry et al., 2018, we also found here that Diptera are especially well-represented in GenBank [15]. When we look at the quality of the COI freshwater records in the NCBI nucleotide database, we found that ~ 43% were fully identified to the species rank.

It is notable how large a proportion of endangered animal species lack COI representation. It is possible that these species are represented in GenBank by other popular markers such as ITS or SSU rDNA [1]. As large scale projects such as iBOL (International Barcode of Life) progresses, it is important that efforts are directed towards building reference libraries for groups such as endangered species. As public databases are updated with new records reanalyzing older data should improve the proportion of metabarcodes that are identified including those of endangered animal species.

### Geographic representation

Having a reference database that represents local species is important for making reliable COI metabarcode assignments. As shown by the Curry et al., (2018) as well as the current study, a good proportion of GenBank COI records represent Canadian freshwater biomonitoring taxa of interest. This is in line with our observation that records disproportionately represent Canadian samples. In this study we also showed that COI records for endangered animal species mostly represent Australia, the United States, and China with records from parts of South America, Africa, and Asia lacking.

For studies outside of Canada, or for applications other than freshwater biomonitoring, an assessment of whether target taxa are represented in current databases (BOLD or GenBank) should be performed prior to conducting taxonomic assignments. Where there are gaps in the reference dataset an attempt should be made to fill these gaps with targeted DNA barcoding of local specimens. Taxa that remain unidentified at finer taxonomic ranks should be acknowledged and could be summarized to more inclusive ranks that are better represented in the database. In molecular ecology studies, a common assumption is that the identifiable portion of the sample is reasonably representative of the full sample. To avoid making this assumption, one can still distinguish broad ecological patterns and detect potential indicators when working directly with ESVs (or OTUs) to avoid excluding unassigned taxa [28,29]. Understanding current database composition and where the gaps exist can help guide future work, for example, by targeting local DNA barcoding efforts, interpreting the results from bioinformatics pipelines, choosing the level of reported taxonomic resolution, and determining statistical confidence for taxonomic assignments [13].

Specific taxonomic gaps in COI GenBank data have been previously published [13–15] and is beyond the scope of this study. Here we highlight both the global distribution of COI records in GenBank, as well as the variability in geographic representation for different subsets of the data, for example, with freshwater taxa of interest versus endangered animal species. We show here that some areas of the world known to have very high endemic diversity, such as in the tropics [30,31], are disproportionally under-represented by fully identified COI records.

### BOLD data in the NCBI nucleotide database

COI records in BOLD and the NCBI nucleotide database are not fully synced or consistently cross-referenced. We show here that a significant portion of the COI records in GenBank, 718,714 (~ 28%), have been flagged with the BARCODE keyword. These records represent 13–15% of COI sequences retrieved from BOLD. We found that a further 33–48% of BOLD records are associated with GenBank records but that these GenBank records are inconsistently cross-referenced with BOLD records (lacking the BARCODE keyword, or cross referencing information placed in the comments field) or they have been subsequently suppressed in GenBank for technical reasons. If the community could improve the cross-referencing of BOLD and GenBank records this could facilitate the re-usability of COI records across studies.

37–55% of BOLD records may be unique to BOLD. It is this subset of the BOLD data that users who create custom databases don’t want to miss. Users are often faced with the choice of using either BOLD or INSDC data for identification by creating custom COI databases to permit high-throughput identifications. The recently published BOLD_NCBI_MERGER script helps to combine records from BOLD with those in GenBank for use with BLAST and MEGAN lowest common ancestor taxonomic assignment [32]. The tool helps to combine high quality COI barcode records from BOLD with the broader taxonomic coverage of COI records from the NCBI nucleotide database. This approach would help assign taxonomy to non-metazoan taxa often present in metabarcoding and metagenomic datasets that could be informative for biomonitoring analysis [33,34]. Future taxonomic assignment method developments would likely benefit from combining these databases to improve overall COI record representation.

### Minimum information about marker gene sequences (MIMARKS)

The Genomics Standards Consortium (GSC) has already outlined recommendations for the minimum information about a marker gene sequence (MIMARKS) that should be submitted with released sequences [35]. That study indicates which metadata fields should be mandatory or environment-specific. Whenever possible, values are based on a controlled vocabulary or ontology. Major databases such as BOLD and GenBank already support these standards. We show here that across the COI Eukaryote NCBI nucleotide records 74% have country and 51% have latitude-longitude metadata (part of MIMARKS). In contrast, nearly all GenBank BARCODE records have country and latitude-longitude metadata. If the community could further improve their compliance with MIMARKS this could greatly contribute to the re-usability of COI GenBank data across studies.

### Incorporating COI references into existing bioinformatic pipelines

The significance of metabarcoding for ecology and biomonitoring more broadly have been shown [36]. COI metabarcoding has its roots in the COI barcoding initiative as well as the long history of metagenomic marker gene surveys for microbial ecology investigations [37–39]. Reference databases such as SILVA and GreenGenes for 16S rDNA as well as UNITE for ITS rDNA [40–42] have been integrated into popular bioinformatics pipelines such as MOTHUR and QIIME2 (https://qiime2.org/) [43,44]. For the COI marker, the most commonly used reference sets come from BOLD [10] and additional curated COI reference sets mined from GenBank are available [13,45].

To enable COI sequences to be analyzed along-side other popular metabarcoding markers, future work should make curated COI reference sets available through the same popular bioinformatic pipelines already widely adopted by the broader molecular ecology community. First steps in allowing COI resources to be integrated with more general bioinformatics pipelines are being developed. The BOLD_NCBI_MERGER script discussed above allows this reference set to be used with microbiome analysis tool MEGAN Community Edition [32,46]. Recent work also provides a curated COI reference set that can be used with the RDP classifier [13,47]. The MIDORI web server also facilitates the classification of mitochondrial markers, including COI, with different methods including the popular RDP classifier and the USEARCH SINTAX classifier [48]. Curated, comprehensive (BOLD + GenBank), and updated COI reference sets should be made available in formats to allow these to be used with popular taxonomic assignment methods.

### The problem of insufficiently identified sequences: Hidden opportunities

It is not uncommon for current COI metabarcode studies to only identify a fraction of the total number of sequences, operational taxonomic units, or exact sequence variants. It has been assumed that the remaining sequences represent a mix of sequence artefacts (non-specific amplification products, chimeric sequences, sequencing errors, etc.) and real species that remain insufficiently identified due to a lack of representatives in reference databases. We have shown in this study that the intersection of COI sequences in BOLD and GenBank is relatively small and this could be one possible reason for insufficiently identified records. On the other hand, if insufficiently identified sequences represent existing named species not present in any public database, then sequencing type specimens should improve taxonomic assignment rates. Similar initiatives have already been initiated for prokaryotes and fungi [49–51]. If, however, insufficiently identified sequences from metabarcode studies represent new taxa, this implies that metabarcoding studies may also be an important new tool for local species discovery as has been found for prokaryotes and fungi [52,53]. This distinction is important because until now COI metabarcoders have been consumers of taxonomic information, such as the high quality records provided by BOLD. It may now be possible for taxonomists to turn the table on this relationship and mine metabarcode data for novel species. In conjunction with non-destructive sampling methods, vouchered bulk samples (e.g. from benthic kicknets) could harbor intact new specimens for formal description using more traditional methods [54,55]. Geotagged records could also guide taxonomists on where to search for novel local taxa.

Another way to handle insufficiently identified sequences, for example from eDNA and mixed community studies, would be to integrate them with existing fully identified sequences. With fungal ITS rDNA, for example, an increasing proportion of insufficiently identified sequences was documented along-side the rise in use of DNA-based methods for ecological studies [56,57]. The explosion in insufficiently identified fungal ITS rDNA sequences effectively out-paced the ability for traditional taxonomy to study and name all new species. Instead, the disambiguation of insufficiently identified sequences was addressed by developing species hypotheses (SH) [29]. The SH concept is similar to a COI Barcode Index Number (BIN) in that it is a cluster of similar sequences, and each SH is given a stable numeric label in the UNITE database. Fungal SH’s takes the BIN concept a step further by clustering insufficiently identified sequences from environmental samples into clusters with stable naming to allow cross-referencing across other metabarcode studies.

A known issue with clusters, however, is that the composition can change depending on order of sequences in a file (when using greedy clustering methods) or by the clustering algorithm chosen (single-, complete-, average-linkage). A method developed to overcome these issues to generate stable operational taxonomic units (OTUs) is SWARM [58]. In any case, users are left with the difficulty of interpreting their clusters as they may not represent unique species. In this study, we show that the rate of insufficiently identified COI records deposited to GenBank is increasing faster than the rate of fully identified records. Looking forward, we must as a community find a realistic way to integrate fully identified as well as insufficiently identified COI records from all sources including COI barcodes as well as sequences from eDNA and mixed community studies.

An emerging practice in the metabarcoding/marker gene community has been to move away from working with sequence clusters and instead focus on exact sequence variants (ESVs) [59]. An ESV can be thought of as an OTU defined by a sequence similarity cutoff of 100%. When using an OTU-based approach, errors generated during PCR and sequencing are absorbed into the cluster. When using an ESV-based approach it therefore becomes essential to denoise new sequence data using a method appropriate for the sequencing platform, such as USEARCH-unoise3 or DADA2 for Illumina MiSeq data, and exclude rare ESVs [60–63]. ESVs have a more straight-forward interpretation than OTUs, which can facilitate easier combinability across studies. New COI sequences can simply be mapped to existing ESVs with 100% sequence similarity and remaining unique sequences become new ESVs. Another benefit is improved resolution by avoiding the accidental ‘lumping’ of ESVs from different species into single clusters [64]. We can envisage how COI ESVs, generated from individual specimens as well as eDNA and mixed community studies, could be combined with a stable numbering system to allow for standardized cross-referencing. Such a method would allow for the detection of biodiversity at a finer level of resolution, capturing sequence-level variation and geographic patterns that would otherwise be obscured in BIN clusters.

We have demonstrated the growth of COI reference records over the past 15 years. We have emphasized the importance of including geographic metadata with COI sequences deposited to the INSDC. Growth in the adoption of COI metabarcoding applications has been substantial in recent years making high quality public COI reference databases an important resource across many fields for years to come.

## Supporting information

S1 Fig The number of COI GenBank records for freshwater biomonitoring target taxa in the NCBI nucleotide database are biased towards Diptera. (PDF)

S2 Fig The number of COI GenBank records deposited in the nucleotide database has grown since 2003. A) The COI barcoding initiative was first introduced by Hebert et al. (2003) and the first COI records flagged with the BARCODE keyword were deposited in 2004 [25]. B) The number of records deposited for freshwater biomonitoring target taxa were tracked from 2003 to 2017. C) The number of records that represent IUCN endangered species were tracked from 2003 to 2017.
(PDF)

S3 Fig Fully identified COI GenBank records have a global distribution that varies according to data partition. The number of records per country, where this data is available, is shown in the legend (log scale): A) BARCODE, B) Freshwater, C) IUCN endangered species. Latitude-longitude data, where this data is available, is plotted as points in ‘orange’ for BARCODE records, in ‘turquoise’ for freshwater records, and in ‘red’ for endangered animal species.
(TIFF)
