# Supplementary figures and images for: Over 2.5 million COI sequences in GenBank and growing
Source: PLoS One. 2018 Sep 7;13(9):e0200177. doi: 10.1371/journal.pone.0200177 (PMC6128447; doi:10.1371/journal.pone.0200177)

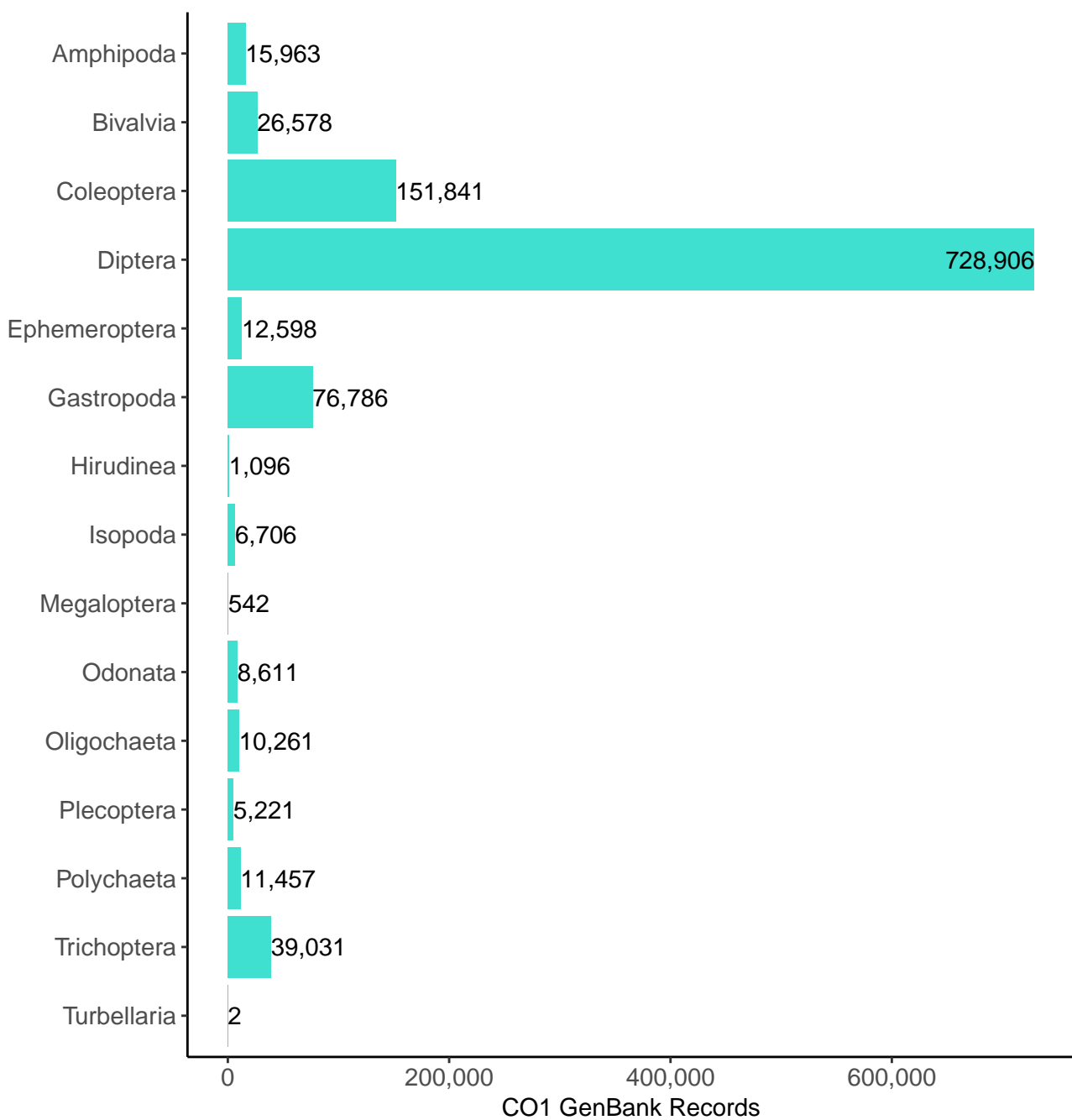

Supplement: S1 Fig — (PDF) [file pone.0200177.s001.pdf]

a) BARCODE

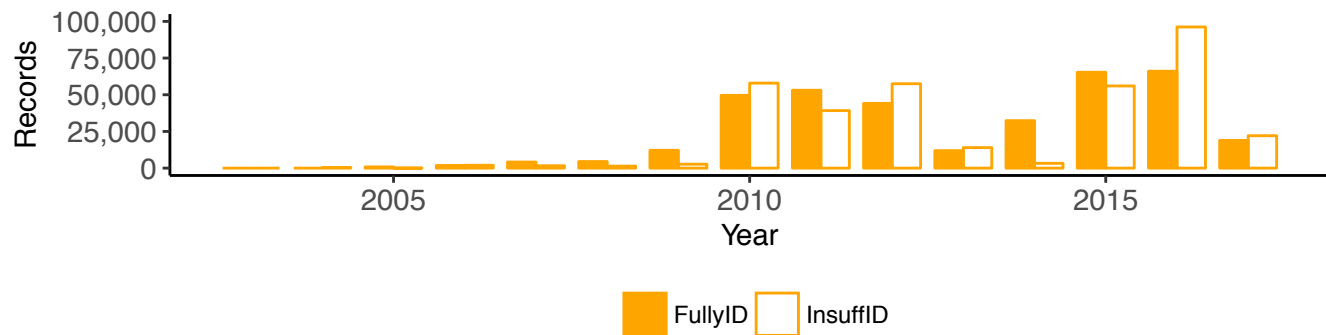

b) Freshwater

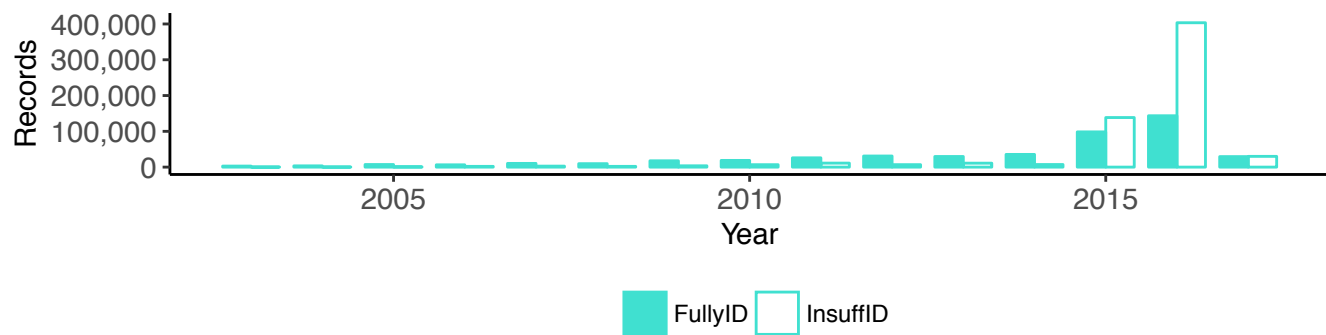

c) Endangered species

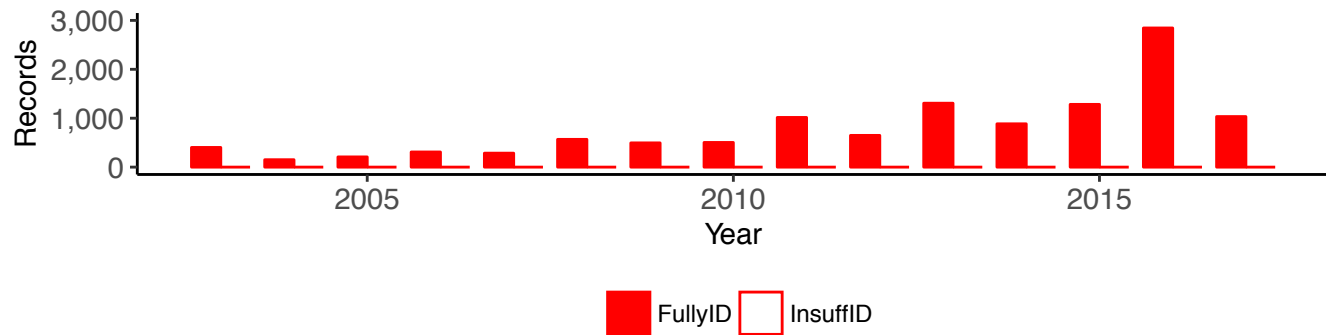

Supplement: S2 Fig — A) The COI barcoding initiative was first introduced by Hebert et al. (2003) and the first COI records flagged with the BARCODE keyword were deposited in 2004 [25]. B) The number of records deposited for freshwater biomonitoring target taxa were tracked from 2003 to 2017. C) The number of records that represent IUCN endangered species were tracked from 2003 to 2017. (PDF) [file pone.0200177.s002.pdf]

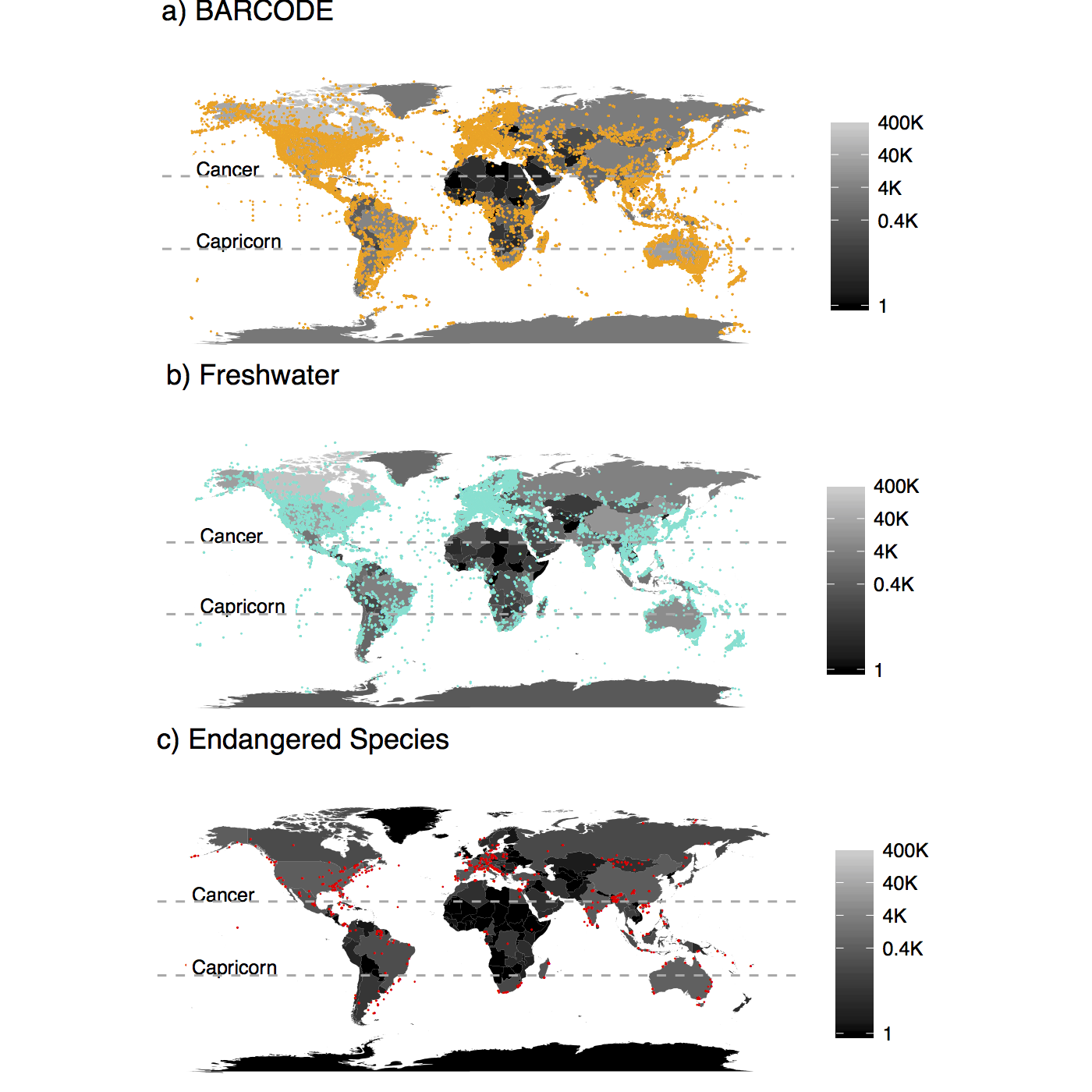

Supplement: S3 Fig — The number of records per country, where this data is available, is shown in the legend (log scale): A) BARCODE, B) Freshwater, C) IUCN endangered species. Latitude-longitude data, where this data is available, is plotted as points in ‘orange’ for BARCODE records, in ‘turquoise’ for freshwater records, and in ‘red’ for endangered animal species. (TIFF) [file pone.0200177.s003.tiff]
